# Supplementary material for: The Sulfide-Responsive SqrR/BigR Homologous Regulator YgaV of Escherichia coli Controls Expression of Anaerobic Respiratory Genes and Antibiotic Tolerance
Source: Antioxidants (Basel). 2022 Nov 28;11(12):2359. doi: 10.3390/antiox11122359 (PMC9774250; doi:10.3390/antiox11122359)
Supplement: Supplementary file 1 [file antioxidants-11-02359-s001.zip › Supplemental_Table&Figs.pdf]

Supplemental Table S1 MRM parameters of iCOPS for YgaV and GS-S-SG

Analytes	Precursor ion (m/z)	Product ion (m/z)	Cone voltage (V)	Collision voltage (V)	Polarity
ILCML	592.3	104.0	30 or 60	30.0	+
KNVYCP	362.2	136.2	30 or 60	40.0	+
ILCML-KNVYCP disulfide	438.2	136.2	30 or 60	40.0	+
ILCML-KNVYCP trisulfide	448.9	136.2	30 or 60	40.0	+
ILCML-KNVYCP tetrasulfide	459.6	136.2	30 or 60	40.0	+
ILCML-KNVYCP pentasulfide	470.3	136.2	30 or 60	40.0	+
GS-S-SG	645.1	387.0	5 – 95	21.0	+
GSH	308.3	179.0	5 – 95	21.0	+
GSSH	340.4	211.0	5 – 95	21.0	+

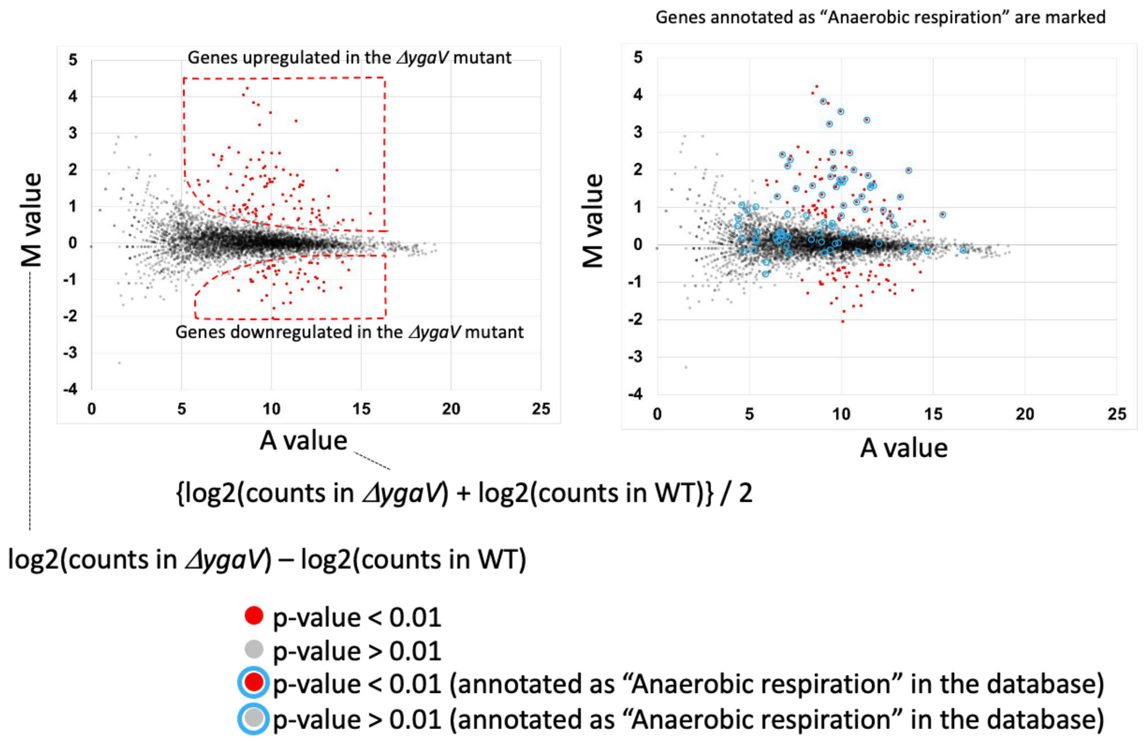

Supplemental Figure S1 The MA plot constructed with the RNA-seq data of *E. coli* WT and *ygaV* mutant.

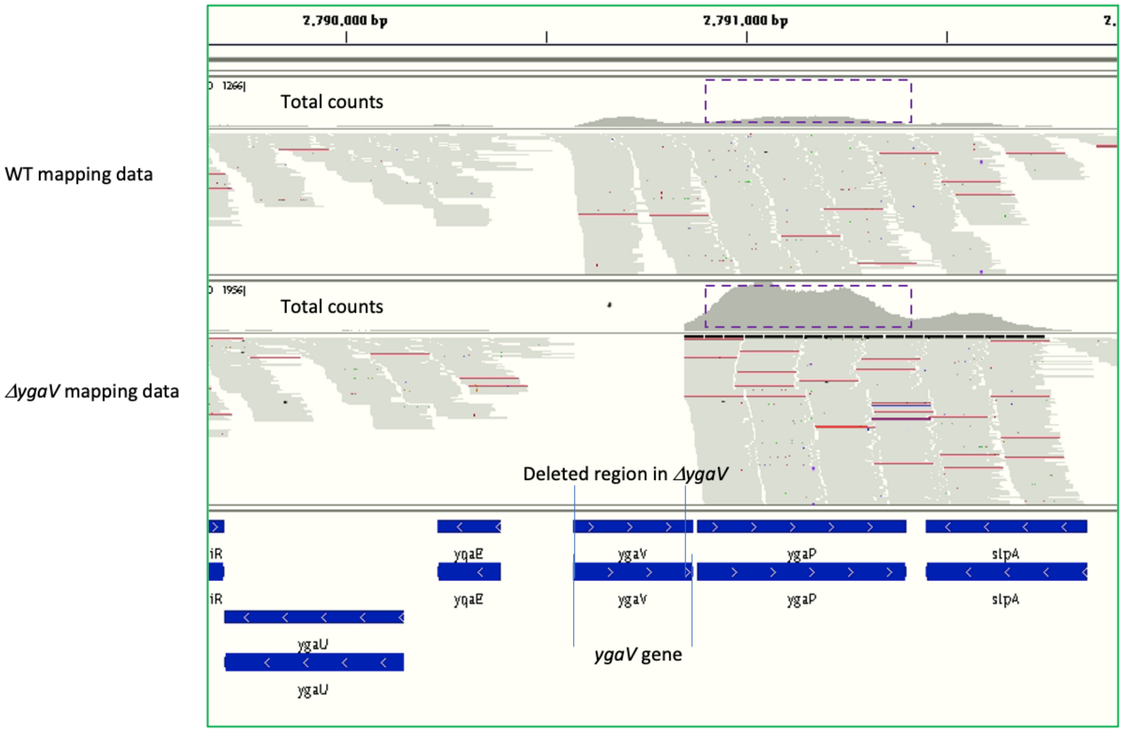

Supplemental Figure S2 Expanded mapping features around the *ygaVP* operon region of the RNA-seq data of the *E. coli* WT and *ygaV* mutant. Increased transcripts of *ygaP* in the *ygaV* mutant compared to that in WT are highlighted by purple dashed boxes.

```
                                                            Cys31
YgaV   -----------MTELAQLQASAEQAAALLKAMSHPKRLLILCMLSGSPGTSAGELTRITG
SqrR   -MGSDTDERCAALDAEEMATRARAASNLLKALAHEGRLMIMCYLASGE-KSVTELETRLS
BigR   MVNEMRDDTRPHMTREDMEKRANEVANLLKTLSHPVRLMLVCTLVEGE-FSVGELEQQIG
                       ::   *: .  :*::: *  **:::* *  .  *. **     .

                                                                 Cys98
YgaV   LSASATSQHLARMRDEGLIDSQRDAQRILYSIKNEAVNAIIATLKNVYCP-----
SqrR   TRQAAVSQQLARLRLEGLVQSRREGKTIYYSLSDPRAARVVQTVYEQFCSGD
BigR   IGQPTLSQQLGVLRESGIVETRRNIKQIFYRLTEAKAAQLVNALYTIFCAQEKQA
          .: **:*. :*:.*::.::*: : : * : :  .   :  :  ::*
```

Supplemental Figure S3 The amino-acid sequence alignment of the *E. coli* YgaV, *R. capsulatus* SqrR and *X. fastidiosa* BigR. Accession numbers are P77295 (UniProt), D5AT91 (UniProt) and Q9PFB1 (UniProt), respectively. Identical and similar amino acids were marked by asterisks and periods or colons, respectively.

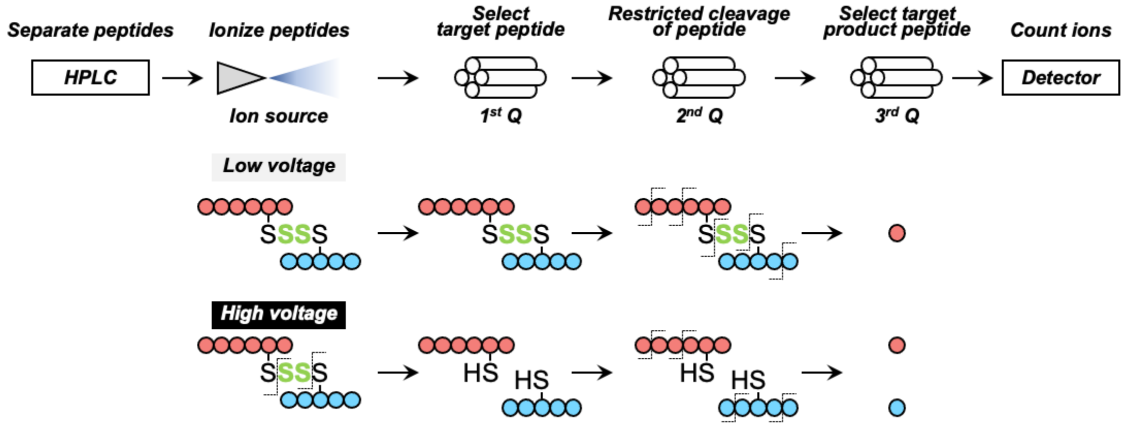

Supplemental Figure S4 Scheme of Ionization-associated clevage of oxidized polysulfur (iCOPS) method. After separation by high performance liquid chromatography (HPLC), peptides are transferred into the ion source. Under low voltage ionization conditions, peptide complex with intra-oxidized polysulfur structure is ionized as the intact form, then detected in the multiple reaction monitoring (MRM) mode. On the other hand, a high voltage ionization causes the cleavage of the intra-oxidized polysulfur bridge between peptides, resulting in the release of peptides containing a free thiol group, which can be simultaneously detected at the same retention time. For more details, see Materials and Methods section of the text.

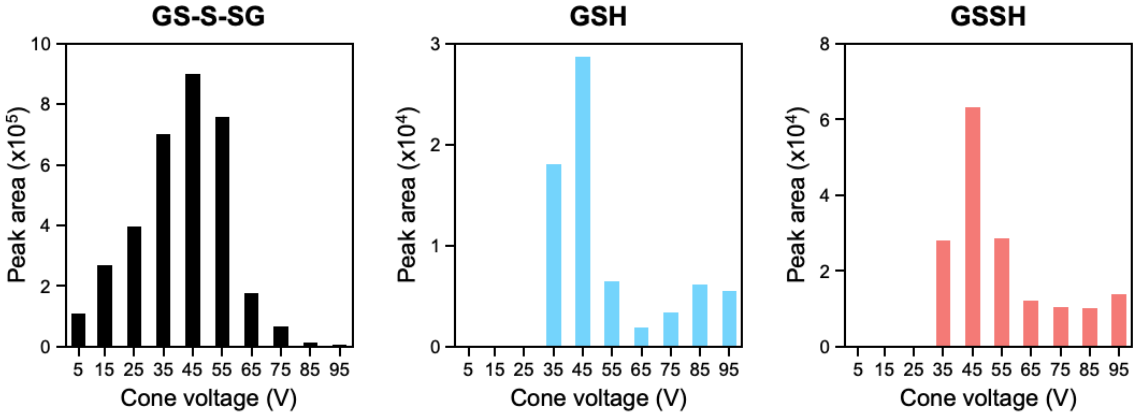

Supplemental Figure S5 Ionization-associated cleavage of oxidized polysulfur on oxidized glutathione trisulfide. Oxidized glutathione trisulfude (GS-S-SG) was used as a model polypeptide containing intra-oxidized polysulfur bond and analyzed for the evaluation of iCOPS method. 10 μM GS-S-SG was chromatographycally separated and ionized in the ion source with various cone voltage sets from 5 V to 95 V, followed by the MRM detection specific for intact GS-S-SG as well as GSH/GSSH formed by the in-source fragmentation. There was no detectable signal of GSH and GSSH formed by in-source fragmentation of GS-S-SG with the cone voltage set below 25 V. On the other hand, the formation of GSH and GSSH was observed at the cone voltage set above 35V, but ionization with the cone voltage set higher than 55 V caused the intense fragmentation of GS-S-SG, resulting in the decrease in GSH and GSSH yield.

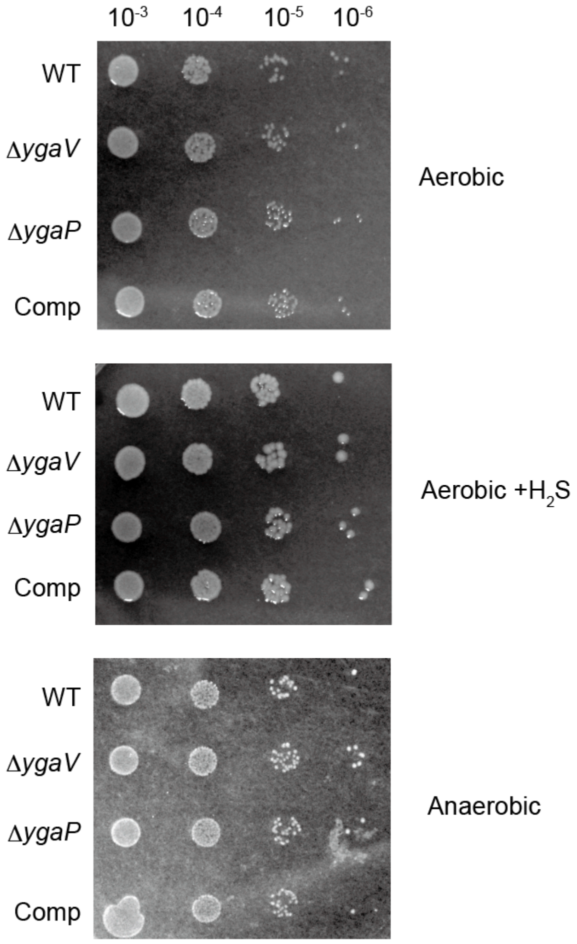

Supplemental Figure S6 Growth of *E. coli* WT, Δ*ygaV*, Δ*ygaP* and *ygaV* complementing strain (Comp) grown under aerobic, aerobic H_2S atmospheric and anaerobic conditions.

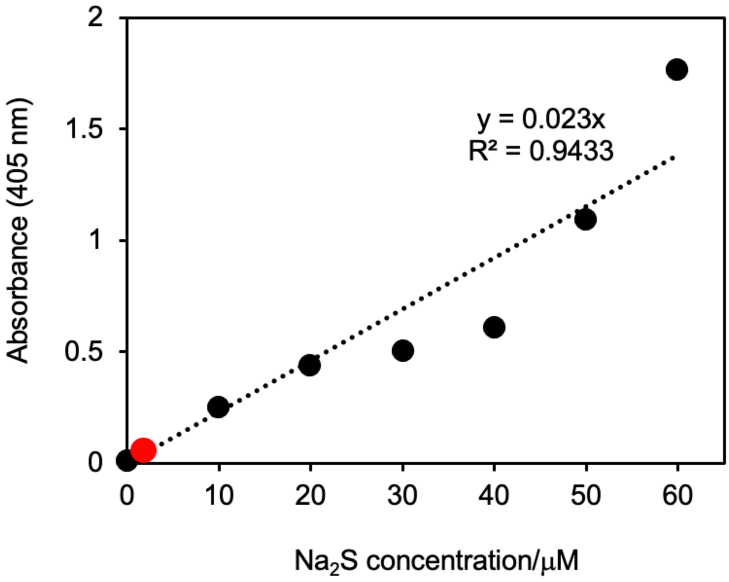

Supplemental Figure S7 Estimation of sulfide levels in the LB medium incubated in the gaseous H_2S-atmospheric conditions. The calibration curve was prepared with 10, 20, 30, 40, 50 and 60 µM Na_2S solution, according to the manufacturer's instruction of the OxiSelect Free Hydrogen Sulfide Gas Assay Kit (Cell Biolabs) (black circles). The red circle indicates the value of the LB medium incubated in the H_2S-atmospheric conditions.

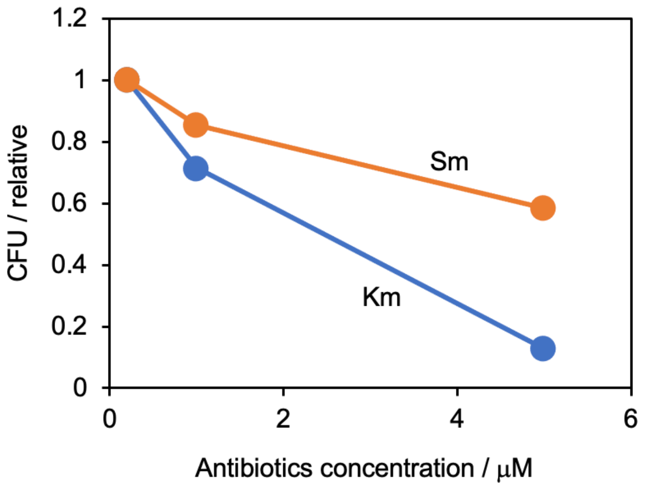

Supplemental Figure S8 Relative colony-forming-unit (CFU) of *E. coli* WT incubated with 0.2, 1.0 and 5.0 μg ml^{-1} kanamycin (Km) or streptomycin (Sp) for 30 min. CFU of 0.2 μg ml^{-1} Km or Sm was set as 1.
